# A Rare Case of Cecal Adenocarcinoma Presenting as Intussusception

**DOI:** 10.7759/cureus.48899

**Published:** 2023-11-16

**Authors:** Nismat Javed, Margaret Dente, Haider Ghazanfar, Abhilasha Jyala, Ariyo Ihimoyan

**Affiliations:** 1 Internal Medicine, BronxCare Health System, Icahn School of Medicine at Mount Sinai, New York, USA; 2 Internal Medicine, American University of the Caribbean School of Medicine, Cupecoy, SXM; 3 Internal Medicine, Bronxcare Health System, Bronx, USA; 4 Internal Medicine, BronxCare Health System, Bronx, USA; 5 Gastroenterology, BronxCare Health System, Bronx, USA

**Keywords:** prognosis, management, colorectal cancer, intussusception, cecal adenocarcinoma

## Abstract

Colorectal cancer is widely recognized as one of the most common types of cancer worldwide. The management and outlook for colorectal cancer depend on its specific characteristics and how it presents clinically. Despite the identification of various risk factors and causes, cecal carcinoma, a type of colorectal cancer, is infrequent in Western populations under 50 years of age, and little research has been conducted on its epidemiology. Additionally, intussusception, a medical condition where one part of the intestine slides into another, is relatively rare among younger individuals. In this case report, we present a 36-year-old male who presented with abdominal pain. A physical exam revealed mild right-sided and peri-umbilical tenderness. A computed tomography scan of the abdomen and pelvis with contrast revealed long segment intussusception involving the terminal ileum and cecum. The patient underwent a reduction of intussusception and hemicolectomy. He was diagnosed with invasive cecal adenocarcinoma with metastasis to lymph nodes. He was started on chemotherapy and has been following as an oncology outpatient.

## Introduction

Colorectal cancer (CRC) is widely recognized as one of the most prevalent neoplastic malignancies globally, carrying an increasing burden of morbidity and mortality [1]. While there has been an overall decrease of approximately 2% per year in CRC mortality in the United States over the last decade, there has been a concerning increase in mortality among patients under 50 years of age, rising by 1.2% annually, and these individuals are often diagnosed at more advanced stages [2]. Particular emphasis should be placed on right-sided colorectal cancers (RCC). These cancers have a different embryological origin compared to left-sided CRC and are associated with a notably worse prognosis [3]. RCC is more common in women and is linked to specific risk factors, including high consumption of polyunsaturated fat, trans-fat, cholesterol, and lactose [4]. Despite extensive screening efforts, isolated cases of cecal cancer are relatively rare, primarily due to its distinct morphological characteristics [5]. Intussusception in adults, a phenomenon where a segment of the bowel slides into another, is a rare occurrence, constituting merely 5% of all intussusception cases and ranging from 1% to 5% of instances of adult bowel obstructions [6]. When a lead point pathology is identified, it often indicates diffuse metastatic disease, including melanoma. However, in adults, ileocolic and colonic intussusception with lead point pathology is most frequently associated with primary malignant adenocarcinoma, especially cecal adenocarcinoma involving the ileocecal valve [7]. The incidence of cecal carcinoma in Western populations among individuals under the age of 50 is rare, and its epidemiology remains poorly studied. While previous cases of cecal adenocarcinoma presenting as intussusception have been reported in older patients, there is no existing knowledge of such cases in younger patients [8]. In this case report, we describe the experience of a 36-year-old male who presented with abdominal pain and was found to have intussusception caused by cecal adenocarcinoma.

## Case presentation

This patient is a 36-year-old African-American man who presented to the emergency department with a one-day history of abdominal pain. The pain was described as peri-umbilical, more to the right, intermittent, and crampy, associated with nausea and some episodes of greenish, foul-smelling loose stools. There were no aggravating or relieving factors associated with the pain. He denied any fever, chills, vomiting, or weight loss. He did not mention any history of prior travel or any contact history. Past medical history was significant for autism, epilepsy, hypothyroidism, and asthma. His home medications included levetiracetam 750 mg, vitamin B12 1000 mcg, cholecalciferol 50,000 international units, montelukast 10 mg, levothyroxine 100 mcg, and albuterol. He had no known drug allergies. The patient had never had surgery or endoscopy, denied ever smoking or using alcohol or recreational drugs, and had no family history of cancer.

In the emergency department, the patient’s vitals included a temperature of 98.6 degrees F, pulse of 86 beats per minute, blood pressure of 138/78 mmHg, respiratory rate of 20 breaths per minute, and oxygen saturation (SpO_2_) of 98% on room air. On physical exam, the patient had mild right-sided and peri-umbilical tenderness but there was no distension, rebound pain, or guarding. Initial investigations, including white blood cell count, liver function tests, and lactic acid, were within normal limits as shown in Table 1.

**Table 1:** Initial laboratory investigations

Investigation	Result	Normal Range
Hemoglobin (g/dl)	14.9	12.0-16.0
WBC (/uL)	7400	4800-10800
Platelets (/uL)	202000	150000-400000
Sodium (mEq/L)	142	135-145
Potassium (mEq/L)	4.4	3.5-5.0
Calcium (mEq/L)	10.3	8.5-10.5
Chloride (mEq/L)	101	98-108
Glucose (mg/dl)	90	70-120
Bicarbonate (mEq/L)	30	24-30
Blood Urea Nitrogen (mg/dl)	9	6-20
Creatinine (mg/dl)	1.0	0.5-1.5
Albumin (g/dl)	4.8	3.4-4.8
Total Bilirubin (mg/dl)	0.4	0.2-1.2
Direct Bilirubin (mg/dl)	<0.2	0.0-0.3
Alkaline Phosphatase (unit/L)	113	53-128
Alanine Aminotransferase (unit/L)	19	5-40
Aspartate Aminotransferase (unit/L)	16	9-48

**Figure 1:**
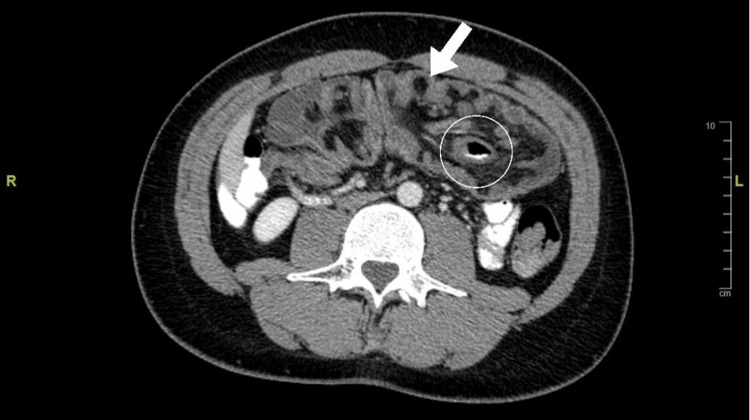
CT scan of the abdomen and pelvis without contrast showing long segment intussusception marked by the arrow and lead point marked by the circle

**Figure 2:**
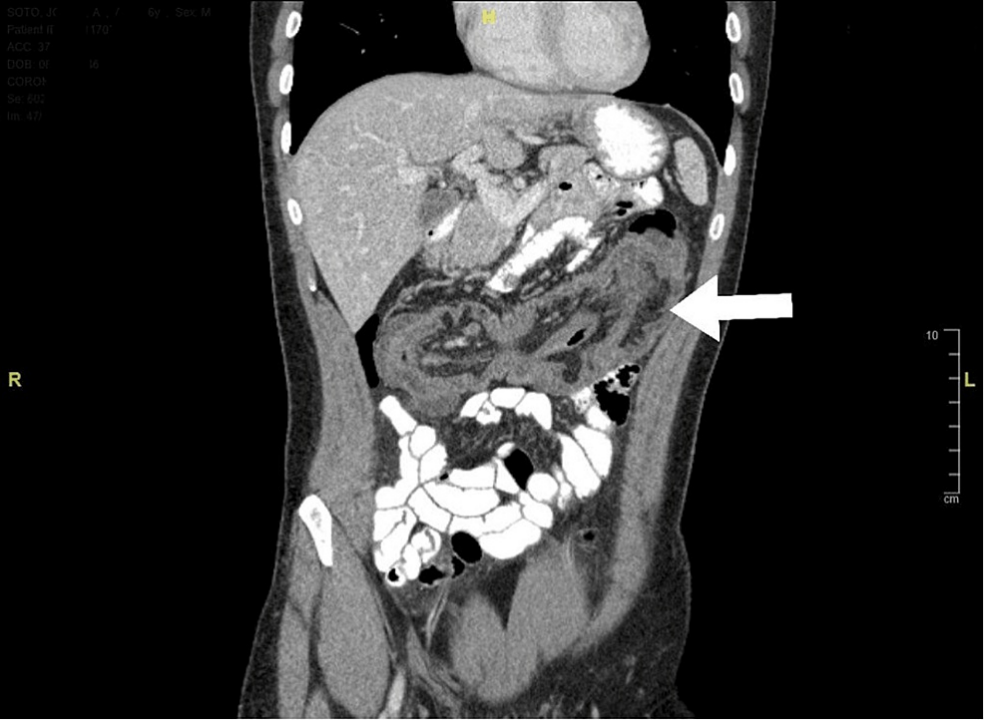
CT scan of the abdomen and pelvis without contrast (coronal view) showing long segment intussusception marked by the arrow

Carcinoembryonic antigen levels were normal. A computed tomography (CT) scan of the abdomen and pelvis with contrast revealed long segment intussusception involving the terminal ileum and cecum passing to the transverse colon, without signs of obstruction (Figure 1 and Figure 2).

Surgery was consulted, and given the high risk of gangrene, the patient underwent immediate exploratory laparotomy and likely bowel resection. The intussusception was reduced and the containing bowel was found to be slightly edematous with a 6 x 5 x 2 cm polypoid, fleshy mass in the cecum, understood to be the lead point. A right hemicolectomy was performed, and pathology revealed invasive adenocarcinoma with moderate differentiation, invading the submucosa. Tubulovillous adenoma was also present. A focus of lymphatic permeation was present. There was metastatic adenocarcinoma in 3/20 pericolic lymph nodes. The margins of the specimen were negative for any malignancy.

The patient recovered without any complications. He was discharged with outpatient oncological and surgical follow-up. His first outpatient oncology appointment was scheduled within 30 days of surgery. The patient had received 12 cycles of FOLFOX (folinic acid, fluorouracil, and oxaliplatin) in the past 11 months. He was scheduled for outpatient gastroenterology follow-up within three weeks of surgery and is due for his next colonoscopy a year later in September 2023, which if negative, will be followed by yearly colonoscopies.

## Discussion

The prompt diagnosis of intussusception in adults can be a challenge due to its rarity and atypical clinical presentation. There are several risk factors that may provide clues about the underlying cause, which can range from benign to malignant lesions or, in a few cases, idiopathic factors. Benign etiologies may include adhesions, adenomas, inflammatory bowel disease, polyps, and submucosal hemorrhages. Malignant pathologies encompass adenocarcinomas, carcinoid tumors, leiomyosarcomas, lymphomas, neuroendocrine tumors, and metastatic carcinomas [7].

Cecal adenocarcinoma presenting as intussusception in adults has been discussed previously as well [9-12]. The presentation is usually in older adults with an age group greater than 65 years of age being commonly involved [9-12]. Males are more likely to be affected as a result [9-11]. The most common presentation is chronic abdominal pain in most of the cases [9-12]. The demographic profile of our case was relatively similar. However, such symptoms in younger African-American patients have not been discussed before [13]. In younger patients, other non-gastrointestinal malignancies, such as non-Hodgkin's lymphoma, have been documented [14]. Most of the cases previously documented had no abnormal lab results except for imaging studies suggestive of the underlying problem. Unlike our case, elevated levels of carcinoembryonic antigen have been observed in one case [9]. Moderately differentiated adenocarcinoma has been commonly observed in histopathological specimens [10,12]. In one case, concomitant tubulovillous adenoma was also observed [10].

CT is the most accurate diagnostic tool, with high sensitivity in detecting intussusception and potential lead points. Various imaging modalities have been discussed in the literature such as abdominal X-rays, ultrasound, CT scans, preoperative colonoscopy, and opaque enemas. Research findings support the diagnostic accuracy of CT scans, ultrasound, opaque enemas, and colonoscopy [15]. However, abdominal X-rays have shown suboptimal diagnostic accuracy. Some reports have indicated that the diagnostic accuracy of CT scans can be as high as 58% to 100% [16,17]. Ciftci et al. also discussed these modalities but noted that ultrasound was less useful for diagnoses [18]. Despite its sensitivity, CT has certain limitations, including accessibility issues, the need for oral and intravenous contrast, and potential delays in diagnosis [19]. Therefore, to prevent delays in diagnosis, clinicians should maintain a high index of suspicion for adult intussusception and consider evaluating risk factors to determine the most appropriate sequence of imaging studies.

Surgical treatment has traditionally been the gold standard for managing intussusception, but a selective therapeutic approach should be considered in patients with underlying malignancies. In some cases with extensive metastasis and post-surgical complications, chemotherapy has also been discussed as a treatment option [20,21].

## Conclusions

In conclusion, intussusception linked to cecal adenocarcinoma is a rare and challenging medical scenario, necessitating a high index of suspicion among healthcare professionals. By considering this diagnosis in adults with nonspecific abdominal pain, medical practitioners can facilitate early detection and treatment, offering patients a more favorable prognosis and improved prospects for recovery.
